# A Three-Step Resolution-Reconfigurable Hazardous Multi-Gas Sensor Interface for Wireless Air-Quality Monitoring Applications

**DOI:** 10.3390/s18030761

**Published:** 2018-03-02

**Authors:** Subin Choi, Kyeonghwan Park, Seungwook Lee, Yeongjin Lim, Byungjoo Oh, Hee Young Chae, Chan Sam Park, Heugjoo Shin, Jae Joon Kim

**Affiliations:** 1 School of Electrical and Computer Engineering, Ulsan National Institute of Science and Technology, Ulsan 44919, Korea; yj2821@unist.ac.kr (S.C.); khpark@unist.ac.kr (K.P.); ohbj87@unist.ac.kr (B.O.); laplume@unist.ac.kr (H.Y.C.); chan4517@unist.ac.kr (C.S.P.); 2 School of Mechanical, Aerospace and Nuclear Engineering, Ulsan National Institute of Science and Technology, Ulsan 44919, Korea; zhffk9@unist.ac.kr (S.L.); yjlim@unist.ac.kr (Y.L.)

**Keywords:** heterogeneous gas, multi-sensor, reconfigurable resolution, readout integrated circuit (ROIC), wireless sensor interface, microelectromechanical (MEMS) device, suspended nanowire

## Abstract

This paper presents a resolution-reconfigurable wide-range resistive sensor readout interface for wireless multi-gas monitoring applications that displays results on a smartphone. Three types of sensing resolutions were selected to minimize processing power consumption, and a dual-mode front-end structure was proposed to support the detection of a variety of hazardous gases with wide range of characteristic resistance. The readout integrated circuit (ROIC) was fabricated in a 0.18 μm CMOS process to provide three reconfigurable data conversions that correspond to a low-power resistance-to-digital converter (RDC), a 12-bit successive approximation register (SAR) analog-to-digital converter (ADC), and a 16-bit delta-sigma modulator. For functional feasibility, a wireless sensor system prototype that included in-house microelectromechanical (MEMS) sensing devices and commercial device products was manufactured and experimentally verified to detect a variety of hazardous gases.

## 1. Introduction

Various gas sensors have been developed for diverse monitoring applications, including air pollution [1] and indoor air quality [2], whose recent applications also extending to biomedical systems, such as an exhaled breath monitoring [3]. Among the many types of gas sensors, solid-state or semiconductor gas sensors have been the preferred technology for Internet of Things (IoT) applications. These sensors in the forms of wireless sensor nodes, thanks to their inherent attributes of low cost, high sensitivity, and miniaturized volume [4]. Moreover, their chemoresistive-type sensing approach provides simplicity in function so that signal conversion and control electronics can be easily combined in the same device. However, their chemoresistance shows wide range of ohmic distribution and different sensor characteristic of sensitivity and selectivity, which depends on target gas kinds [4,5,6]. This necessitates sophisticated sensor interfaces and readout integrated circuits (ROICs) that support a wide range of chemoresistive detection with signal-conversion resolution adequate for target sensitivity and a dynamic range [7]. 

For simple and low-power conversion of resistive sensors, the oscillator-based method [8] that resistance changes are converted to frequency changes in a RC oscillator. Since its resulting frequency is calculated by utilizing a digital counter, its digital conversion resolution has a tradeoff relationship with conversion speed, and both speed and resolution are limited. Another low-power method is the resistance-to-digital converter (RDC) [9], which directly converts the resistance to a digital code. A digital-to-analog converter (DAC) is binary-searched to estimate the sensor resistance through repetitive comparisons. This RDC provides faster conversion speed than in the oscillator-based method, but it requires more complex digital control circuits. For accurate signal conversion of chemoresistive sensors, a conventional expensive method converts the sensor resistance to a voltage and then converts the voltage to a digital code through an analog-to-digital converters (ADC) [10]. In many cases, the resistance to voltage conversion utilizes the Wheatstone-bridge circuit [11], but its linearity is guaranteed only for narrow-range resistance change. Therefore, in order to support wide-range resistance change, it utilizes the resistor DAC for a reference resistance that is programmed to be approximately equal to initial value of the sensor resistance [12]. However, this becomes less accurate at a low resistance range where switch-on resistances are not negligible. Another method is to utilize the current DAC to make the converted voltage of the initial sensor resistance equal to a reference voltage [13], and the chemoresistive change provides a voltage deviation from the reference that is then converted to a digital code in the following ADC. Similarly, in high resistance range where the current DAC value should be very small, leakage current effects increase such that the detection accuracy would seriously degrade.

In order to implement a multi-sensor interface for various kinds of hazardous gases, it is necessary to develop chemoresistive sensors with a wide-range capability. Additionally, the detection resolution needs to be adaptively controlled since the required accuracy changes depend on the kind of gas or its system-operating status. Therefore, this paper presents a reconfigurable multi-gas sensor interface and its ROIC that includes a dual-mode front-end for wide-range resistance-to-voltage conversion and three-step resolution controllability with optimal power consumption. Since electrical characteristics of semiconductor-type gas sensors are affected by ambient temperature and humidity [14,15,16] and their real-time monitoring capability [17] are also included. The ROIC is fabricated in a CMOS process foundry, and in-house microelectromechanical (MEMS) gas sensors with suspended architecture were manufactured and assembled together. A wireless multi-gas monitoring system prototype that includes the ROIC, the in-house MEMS sensors and commercial sensor products is verified to provide real-time detection of several hazardous gases such as CO, H_2_, NO, CH_4_, C_6_H_6_, and SO_2_. 

## 2. Wireless Multi-Gas Sensor Interface

### 2.1. Multi-Sensor System Architecture

**Figure 1:**
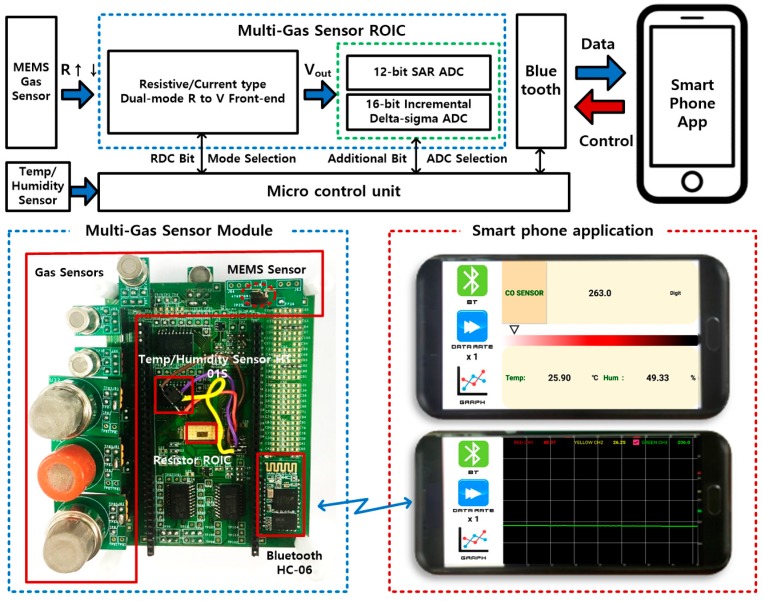
Multi-channel resistive sensor readout Interface with smartphone application.

Figure 1 presents a proposed wireless multi-sensor interface architecture for hazardous gas detection that consists of in-house or commercial sensing devices, a ROIC, a microcontroller unit (MCU), and a Bluetooth module. This multi-sensor interface architecture [18] attributes compactness and flexibility through a reconfigurable interface structure and the migration of signal processing burdens from a sensor tag to a smartphone. Raw sensor data is wirelessly sent to the smartphone where proper calibration and post-processing are performed to reflect each sensor’s characteristics. This proposed multi-sensor interface provides eight resistive sensing channels for in-house or commercial gas sensor devices with characteristic resistances that range from kilo-ohms to mega-ohms. Therefore, a dual-mode readout front-end structure is proposed to support wide-range resistive detection, and a three-step resolution-reconfigurable digital conversion scheme is also proposed to provide an optimal tradeoff of accuracy and power consumption. The dual-mode front-end and reconfigurable conversion are implemented into a single ROIC, and the eight-channel sensor interfaces are provided through the same ROIC by programming reconfigurable readout and conversion modes. For compatibility with various gas sensor devices, seven kinds of commercial gas-sensor device products were included in the system prototype to verify the feasibility of the proposed reconfigurable multi-sensor interface. Since most resistive gas-sensor devices are inherently affected by ambient environment, temperature, and humidity monitoring functions were included together to provide real-time device calibration with post-processing calculations performed on the smartphone. This wireless multi-gas interface was designed to be able to compress or control the amount of real-time monitored data, thus reducing server-side loads in terms of data traffic and storage.

### 2.2. In-House Mems Sensor Device

**Figure 2:**
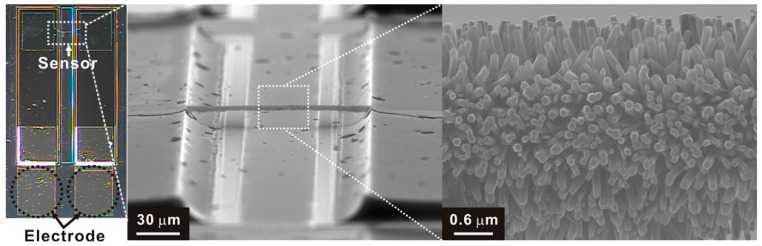
In-house multi-gas sensor based on suspend hierarchical nanowires: (Left) Gas sensor chip, (Center) Suspended hierarchical wire, (Right) ZnO nanowires.

The in-house multi-gas sensing devices are fabricated to have the architecture of a hierarchical suspended wire that connects two posts, as shown in Figure 2. The suspended wire consists of core carbon nanowire (diameter ~ 300 nm, length = 120 μm) and circumferentially grown ZnO nanowires (diameter ~ 55–80 nm, length ~ 1.2–1.6 μm) as a gas sensing material. The suspended carbon nanowire and supporting carbon posts were fabricated monolithically using carbon microelectromechanical systems (MEMS) technology that enables wafer-level carbon nanostructure fabrication using photolithography and pyrolysis [19]. Owing to good mechanical robustness, the suspended carbon nanowire can be selectively coated with gas sensing materials such as Pd nanoparticles and metal-oxide nanowires using electrode position and hydrothermal growth, respectively [20,21]. High aspect ratio and large surface to volume ratio of the suspended wire enables highly sensitive gas sensing. In consideration of the degradation of the gas sensor, a process of etching the silicon substrate to prevent the metal oxide from growing on the silicon substrate at the lower end of the carbon nanostructure was included. 

## 3. Reconfigurable Multi-Gas Sensor ROIC

### 3.1. Dual-Mode Readout Front-End

**Figure 3:**
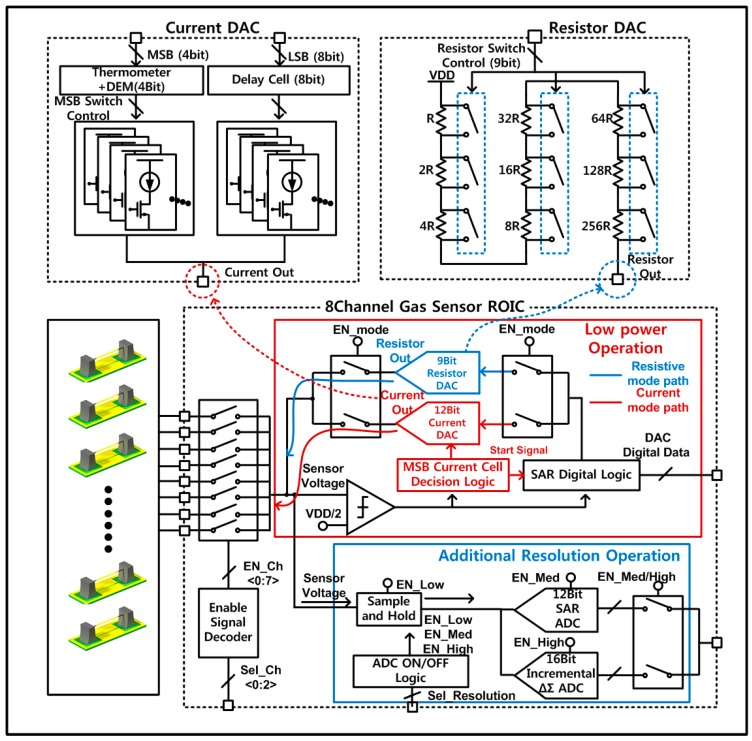
Reconfigurable multi-sensor readout integrated circuits (ROIC) with dual-mode front-end and triple-mode digital conversion.

The proposed ROIC architecture for the wireless multi-gas interface is attributed with two reconfigurable features of dual-mode front-end and three-step digital conversion. Figure 3 shows a detail block diagram of the proposed dual-mode front-end circuit, supporting three-step controllable digital-conversion resolution. The resistor or current DACs together with a sensing resistance constitute two kinds of readout front-end circuits such as the voltage divider (resistive mode) or the resistance-to-voltage converter (current mode). This dual-mode front-end itself can provide low-power RDC function without additional ADCs by implementing the binary resistive search together with a comparator and a successive approximation register (SAR) control logic [9]. For further digital-conversion resolution, the DAC is optimally located around the sensor resistance, and then the front-end works as the resistance-to-voltage converter, resolving the remaining residue by utilizing a low-power SAR ADC [22]. For maximum conversion resolution, a 16-bit incremental delta-sigma ADC [23] is activated instead of the SAR ADC. In this way, the proposed readout front-end is designed to provide the dual-mode operation by utilizing resistive or current DACs. However, the current mode should use very low DAC current to support high-resistance sensors, and then the readout accuracy becomes degraded due to leakage currents or noises from other devices. In case of the resistive mode, low-resistance sensor operation needs small-valued resistance of the resistor DAC, and its resistance accuracy becomes worse due to on-resistance effect of switches inside the DAC that becomes greater as the sensor resistance decreases [13]. 

Therefore, in order to compensate for these regional problems, the proposed dual-mode front-end is designed to use the current mode in low-resistance region and the resistive mode in high-resistance region, sustaining the readout accuracy over wide sensor-resistance region. This dual-mode operation is controlled by programming a control bit of EN_mode, where its activation gives the current mode and its deactivation makes the resistive mode. 

For circuit implementation of the proposed dual-mode front-end, a 12-bit current-steering DAC is adopted for the current mode to support the sensor-resistance range from 250 Ω to 1 MΩ. This current DAC is designed to generate programmable currents from 900 nA to 3.6855 mA and also to have the segmented structure where 4-bit MSB current sources have the thermometer structure and 8-bit LSB current sources have the binary structure. The 4-bit MSB thermometer current sources adopt the dynamic element matching (DEM) to minimize the mismatch effects of current sources [24]. Second, the resistive mode is designed to utilize a 9-bit resistor-string DAC at the front-end to support the sensor-resistance range from 1 MΩ to 10 MΩ. It adjusts the resistor DAC value through the binary research so that the voltage-divider output of the sensor resistance and the resistor DAC becomes close to VCM. The 9-bit resistor DAC has a unit (LSB) resistance of 22.4 kΩ and a total resistance of 11.4 MΩ. This resistive mode is used only for high resistance region greater than 1 MΩ, where the switch on-resistance problem of the DAC is minimized. 

**Figure 4:**
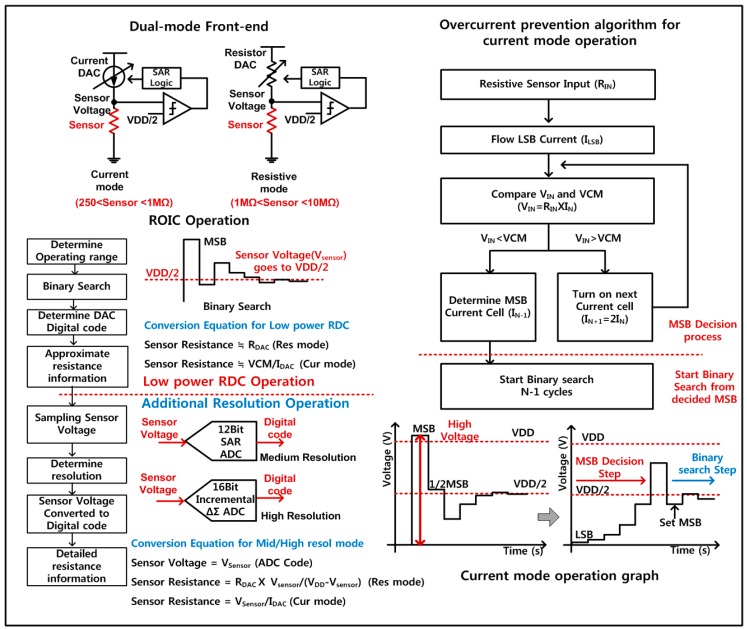
Overcurrent prevention algorithm flow chart and operation graph.

In the current-mode operation, the current DAC is binary-searched so that the output voltage after flowing a sensor resistance is close to a reference voltage. However, when instant sensor resistance is high-valued, this current binary search initially provide high currents, resulting in its instant output voltages higher than the maximum voltage which is allowed for stable operation of semiconductor transistors. Therefore, the proposed current mode is designed to include an over-current prevention algorithm during the binary search of the current DAC. As shown in Figure 4, the over-current prevention algorithm activates only the LSB current of the DAC to give the smallest value initially, and repetitively activates the next higher-bit current source in the DAC with the previous lower-bit deactivated. After the output voltage through the sensor resistance exceeds a reference voltage (VCM), the instant current bit is held on, and the lower bits are decided by utilizing the binary search sequence. Using this proposed over-current prevention search algorithm, it is possible to prevent instant large voltages to give critical damages to transistors and sensor devices. 

### 3.2. Three-Step Reconfigurable Digital Conversion

The proposed ROIC is designed to provide three-step controllability on the tradeoff between the resolution and the power consumption in digital conversion of sensor data. First, the dual-mode front-end itself can work as the low-power RDC, which directly convert sensor resistances to digital data without ADCs. Based on this RDC operation, the current or resistor DAC of the front-end is programmed properly. Then, for higher resolution performances, the front-end converts sensor resistances to voltages, and the resulting voltage outputs are digitized by a 12-bit low-power SAR for medium resolution or a 16-bit incremental delta-sigma ADC for high resolution. In these higher resolution modes, the offset-drift problem of the low-power RDC mode which is mainly to its comparator can be removed or minimized thanks to two-stage structure of the resistance-to-voltage converter and the ADC. In this way, the RDC operation gives the lowest power consumption, and activations of the SAR ADC or the incremental delta-sigma ADC provide medium or maximum resolution, respectively.

**Figure 5:**
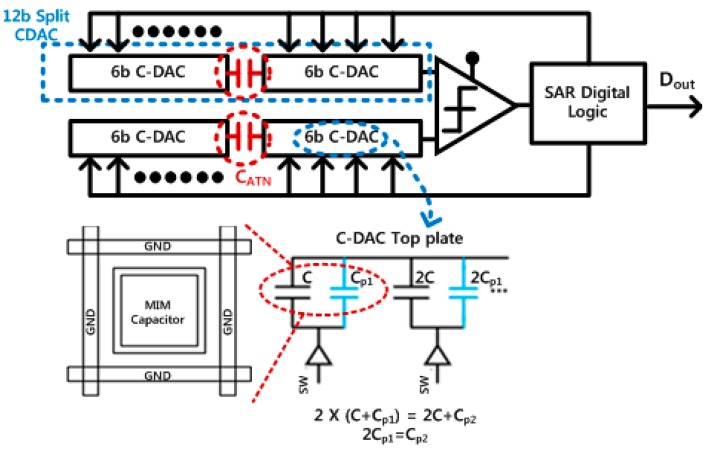
12bit successive approximation register analog-to-digital converter (SAR ADC): (**a**) architecture, and (**b**) unit capacitor layout with parasitic matching.

Figure 5 shows the fully-differential implementation of the 12-bit SAR ADC, which consists of capacitive DACs (C-DAC), a comparator, and a SAR digital logic. The SAR ADC shows inherently low-power characteristics since most sub-blocks perform digital-based operations if a dynamic comparator without standby current is used. For 12-bit implementation of the SAR ADC, two 12-bit C-DACs are required, resulting in relatively large silicon area. Therefore, this work tried to minimize the area of C-DACs by utilizing the split-type structure [25], reducing the area by 16 times when compared with a conventional binary structure. In the split-type C-DACs, parasitic effects on the attenuation capacitor (C_ATN_) might lead to overall performance degradation, and post-layout simulations with parasitic extractions are repetitively performed to achieve the desired resolution. Since the metal-insulator-metal (MIM) capacitor is made up of the top metal and the underlying metal in the layout, and parasitic components, like Cp1 and Cp2, are generated by routing wires in the actual layout. Therefore, the MIM capacitors are ground-shielded to minimize parasitic capacitances from surrounding wires and other capacitors. Additionally, parasitic capacitances of the C-DAC are made efforts to be constant by providing the same adjacent environment.

**Figure 6:**
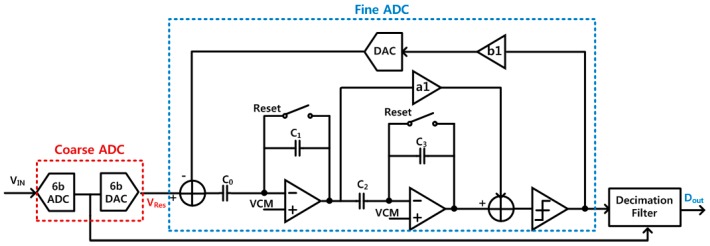
16-bit incremental delta-sigma ADC with two-step structure.

Figure 6 presents detail implementation of the 16-bit incremental delta-sigma ADC for high resolution readout of sensor resistances. It is designed to have a two-step ADC architecture, where the SAR ADC was utilized as a 6-bit coarse ADC and the following feed-forward delta-sigma modulator was used as a fine ADC to analyze the remaining residue of the SAR ADC. This two- step structure requires fewer operation cycles than a conventional incremental delta-sigma ADC, thereby increasing the operation speed and reducing overall power consumption. Also, since internal integrators in the delta-sigma modulator have limited signal swing range, nonlinearity characteristics including gain variation can be greatly reduced. Bit-stream outputs from the delta-sigma modulator are converted into binary digital codes through the decimation filter, achieving the 16-bit digital-conversion resolution. 

## 4. Measurement Results

**Figure 7:**
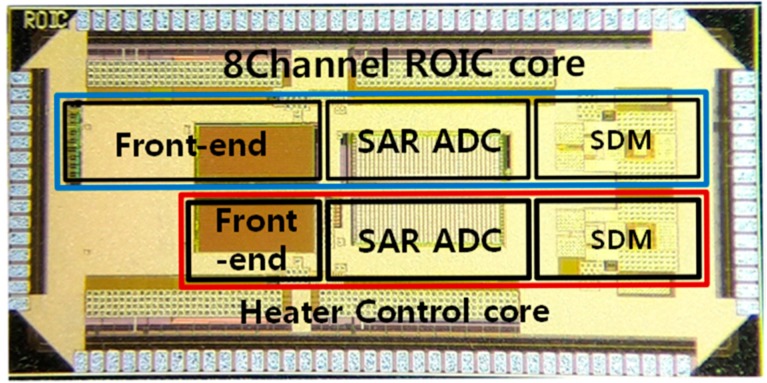
Chip microphotograph of 8-channel gas Sensor ROIC.

**Figure 8:**
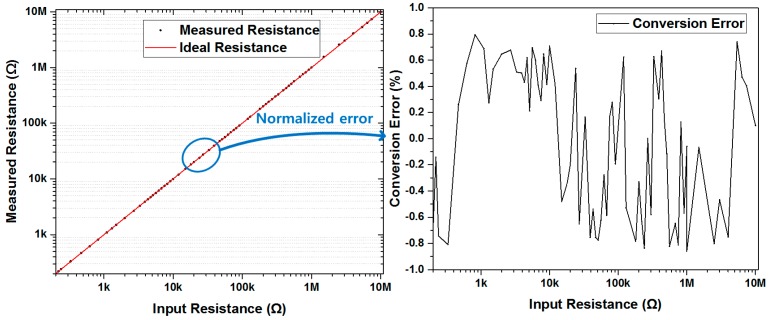
Conversion error measurement results of ROIC in high resolution mode.

**Figure 9:**
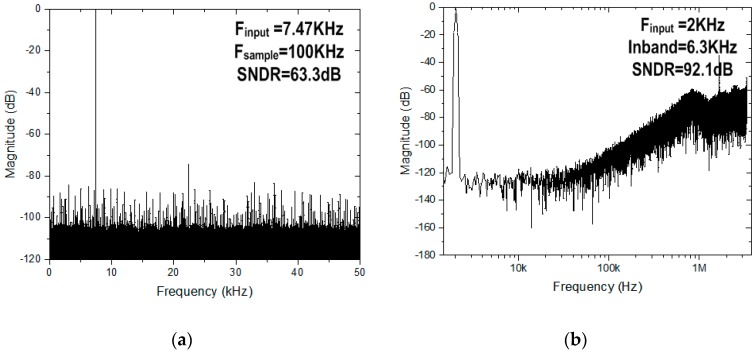
Measured spectrums of (**a**) SAR ADC and (**b**) incremental delta-sigma ADC.

A proposed multi-gas ROIC prototype including the dual-mode front-end and the three-step resolution-reconfigurable digital conversion was fabricated in a 0.18 μm CMOS process. Figure 7 shows a microphotograph of the multi-gas ROIC chip, where the total area is 4 mm × 2 mm and the core size of the ROIC is 2.5 mm × 0.94 mm. This dual-mode ROIC operates in the current mode or the resistive mode, according to characteristic resistance of gas sensors. Figure 8 shows measured ROIC characteristics in the range of 250 Ω–10 MΩ, where the normalized conversion error [12] was included together. The proposed ROIC operates with conversion error less than 0.8% in the medium-resolution mode. For the range of 250 Ω–1 MΩ, the current-mode front-end was used, and the resistive mode was applied for the range of 1 MΩ–10 MΩ. Figure 9 shows measured performances of the 12-bit SAR ADC used for the medium resolution and the 16-bit incremental delta-sigma ADC for the high resolution. The SAR ADC was measured to have the signal to noise and distortion ratio (SNDR) of 63.33 dB with the input frequency of 7.47 kHz and the sampling frequency of 100 kS/s. The incremental delta-sigma ADC achieved 92.1 dB SNDR with the input frequency of 2 kHz and the inband of 6.3kHz, which corresponds to the effective number of bits (ENOB) of 15.3 bits.

The proposed ROIC prototype has reconfigurable capability of resolution and power, and its power consumptions with sub-block breakdown depending on the dual mode and the three-step digital conversion are summarized and compared in Table 1. The RDC operation consumed 57.1 μW–3.14 mW in the current mode and 1.3 μW–13.3 μW in the resistance mode. For the medium resolution operation through the SAR ADC, the total power consumption increased to 1.24 mW–4.43 mW. In the high-resolution operation through the incremental delta-sigma ADC, the best resolution was achieved with power consumption of 1.72 mW–4.86 mW. In this way, through the proposed reconfigurable capability, the power consumption can be optimally controlled depending on the resolution requirement of application systems.

**Figure 10:**
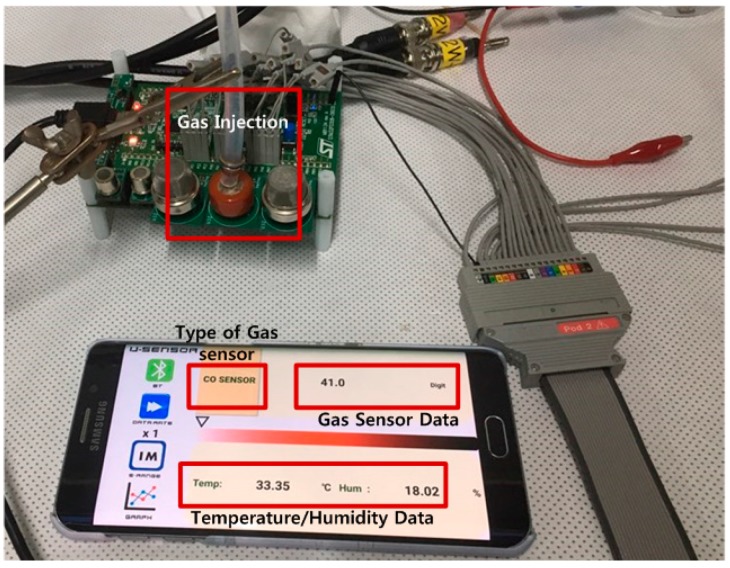
Measurement setup of proposed multi-gas sensor interface.

**Figure 11:**
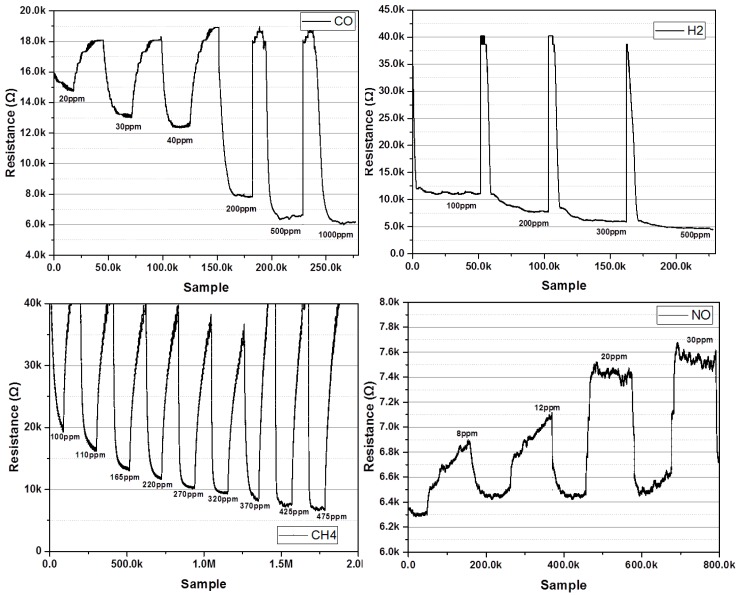
Multi-Gas measurement results of CO, H_2_, NO and CH_4_.

**Figure 12:**
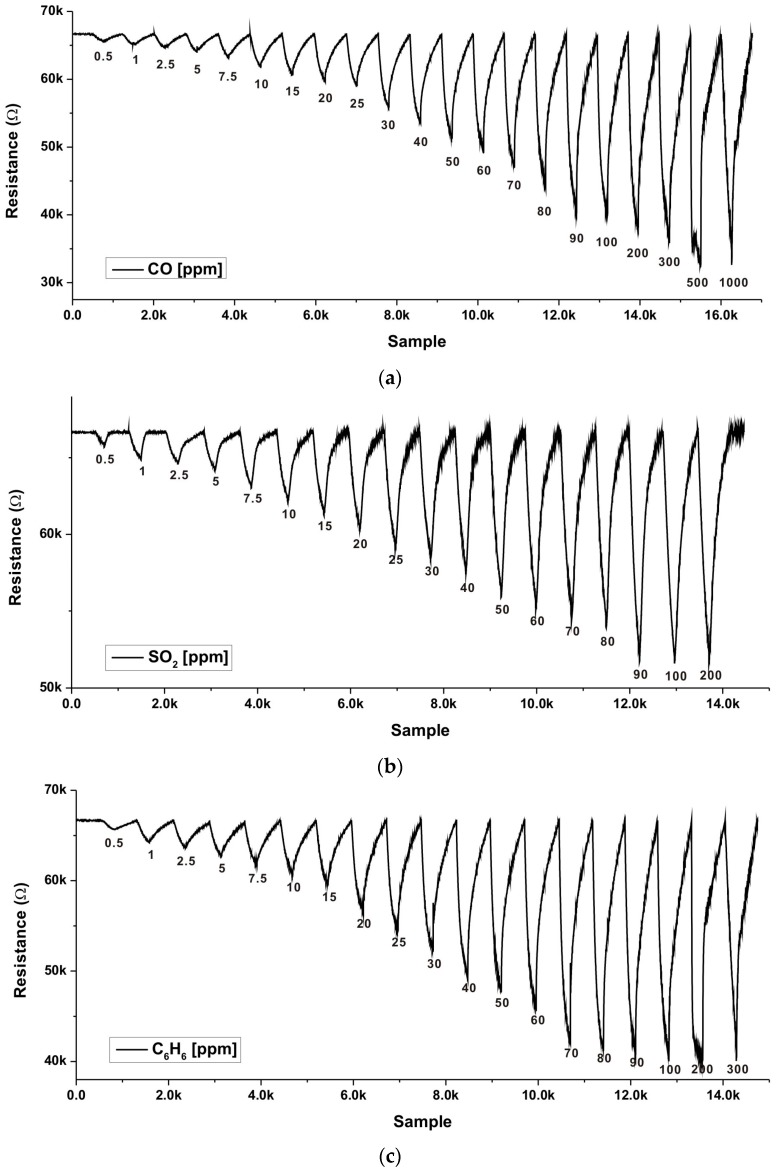
Microelectromechanical (MEMS) Gas Sensor measurement results (**a**) CO (**b**) SO_2_ (**c**) C_6_H_6_.

The proposed multi-gas sensor interface was measured by the test setup, as shown in Figure 10. For gas measurement using commercially available sensors, a multi-gas sensor interface was used to attach an MQ-7 as a semiconductor type CO sensor, GSNT11 as a semiconductor type NO sensor, MQ-5 as a CH4 sensor, and MQ-8 as an H_2_ sensor. Figure 11 shows the results of measuring the commercial gas sensor according to change of gas concentration through the multi-gas sensor interface. The CO gas sensor was measured at concentrations of 20 ppm–1000 ppm, and H_2_ gas sensor was measured at concentration of 100 ppm–500 ppm. The CH_4_ sensor was measured at concentration of 100 ppm–475 ppm, and the NO gas sensor was measured at concentration of 8 ppm–30 ppm. Since each gas sensor has a different resistance and sensitivity, each gas sensor was measured under different concentration conditions. In the gas sensor test for the MEMS sensor, the gas concentration was controlled by mixing the target gas with N_2_ using gas flow meters (GMC1200, ATOVAC, Korea). At each gas concentration, the target gases were injected into a gas sensing chamber for 5 min. Before injecting the target gas and after gas sensing, the gas sensing chamber was purged with N_2_ for 10 min. Figure 12 shows the results of measuring resistance change with the gas concentration by attaching the gas sensor fabricated using MEMS processes to the multi-gas sensor interface. The MEMS sensor is based on a metal oxide nanowire. Therefore, the sensor exhibited gas response to various oxidizing and reducing gases. In this study, we tested the performance of the presented sensor readout interface by measuring concentrations for various gases (CO, SO_2_, C_6_H_6_) using the MEMS sensor. The MEMS sensor exhibited gas responses of 8.1, 7.2, and 10.0% for CO, SO_2_, and C_6_H_6_ at 10 ppm, respectively, as shown in Figure 12. The detailed sensor performances and characteristics of the suspended sensor architecture of the MEMS sensor were reported elsewhere [21]. The resistance change of the gas sensor was measured in the range of 30 kΩ–70 kΩ with changes in the concentration of CO, SO_2_, and C_6_H_6_ gas between 0.5 ppm and 1000 ppm, respectively.

## 5. Conclusions

A reconfigurable multi-gas sensor interface with a dual-mode front-end and three-step resolution controllability was proposed and its system-level functional feasibility was experimentally verified in a wireless system-level prototype. The prototype was designed to support eight channels for chemoresistive sensor devices through a single reconfigurable ROIC, and post-processing calibrations, including temperature and humidity, were performed in a smartphone to minimize load on the sensor module. The designed ROIC prototype was fabricated and its sensor interface was implemented to include in-house carbon MEMS sensor devices and also commercial device products. With further array-type integration work of multiple in-house MEMS devices, this reconfigurable multi-gas interface can be further reduced in size, and power consumption can also be reduced considerably. These characteristics are much suitable for IoT sensor applications.

## Figures and Tables

**Table 1:** Power consumptions of dual-mode ROIC with three-step reconfigurable conversion.

Operation mode	Current mode (250 Ω–1 MΩ)		Resistor mode (1 MΩ–10 MΩ)	
	Component	Power	Component	Power
Low power mode (Front-end)	Current cell (Current flowing in the sensor)	1.4 μW–3.1 mW	Resistor DAC (Current flowing in the sensor)	0.2 μW–12.1 μW
	Reference current cell	54.5 μW	Digital logic + Comparator	1.1 μW
	Digital logic + Comparator	1.1 μW	-	-
	Total (Front-end)	57.1 μW–3.14 mW	Total (Front-end)	1.3 μW–13.3 μW
Medium resolution mode (Front-end + SAR ADC)	SAR ADC	73 μW	SAR ADC	73 μW
	S/H (Amp + CMFB)	1.16 mW	S/H (Amp + CMFB)	1.16 mW
	Total (Front-end + SAR ADC + S/H)	1.29 mW–4.43 mW	Total (Front-end + SAR ADC + S/H)	1.24 mW–1.25 mW
High resolution mode (Front-end + Incremental ADC)	Incremental ADC + S/H	1.72 mW	Incremental ADC + S/H	1.72 mW
	Total (Front-end + Incremental ADC + S/H)	1.78 mW–4.87 mW	Total (Front-end + Incremental ADC + S/H)	1.72 mW–1.73 mW
